# A Compend of the Principles of Homœopathy

**Published:** 1896-11

**Authors:** 


A COMPEND OF THE PRINCIPLES OF HOMŒOPATHY. By Wm. Boericke, M. D., Professor of Materia Medica and Therapeutics at the Hahnemann Hospital College of San Francisco. 160 pages. Price, bound in cloth, $1.50; by mail, $1.60. Published by Boericke & Runyon, 234 Sutter Street, San Francisco. New York: Boericke, Runyon & Ernesty, No. 497 Fifth Avenue.

The book is not only an introduction to the study of Homœopathy for every student, but will be found of helpful service to the generation of young practitioners who have not had the advantage of a systematic training in the principles of Homœopathy. It covers concisely the whole field of Homœopathy, with many references to its literature for the further systematic study of Homœopathy, both as a science and as a practical art.

